# Supplementary material for: Blind Analysis of Food-Related IgG Identifies Five Possible Nutritional Clusters for the Italian Population: Future Implications for Pregnancy and Lactation
Source: Nutrients. 2019 May 17;11(5):1096. doi: 10.3390/nu11051096 (PMC6566756; doi:10.3390/nu11051096)

Frequency distribution of IgG for almond

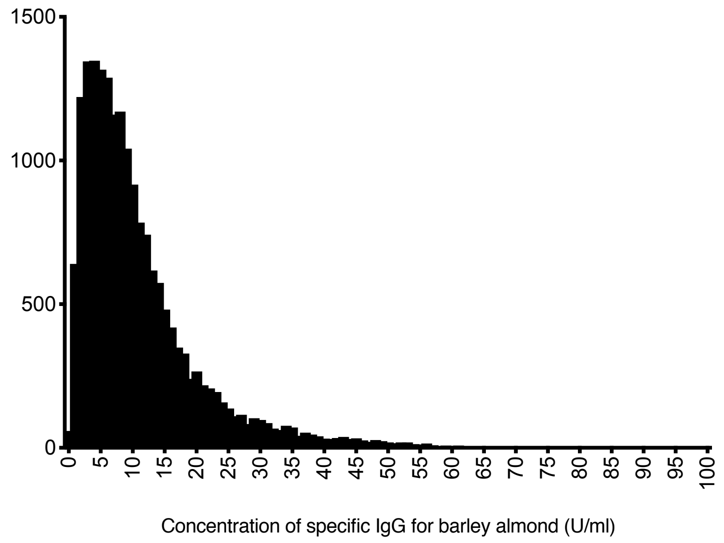

Frequency distribution of IgG for *Aspergillus fumigatus*

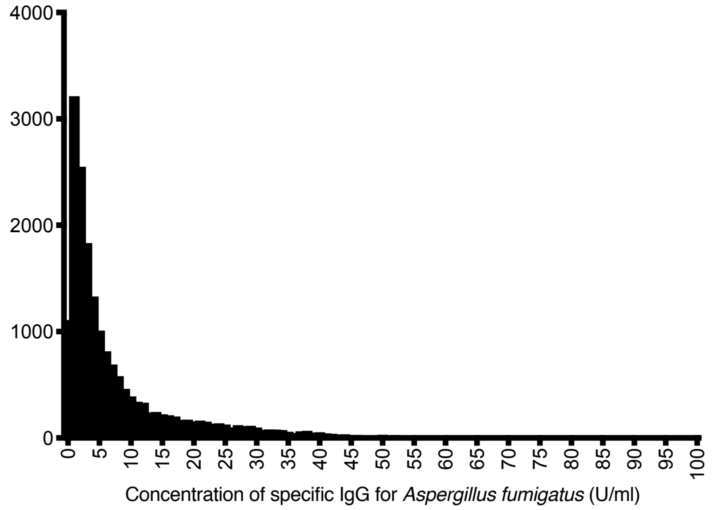

Frequency distribution of IgG for barley

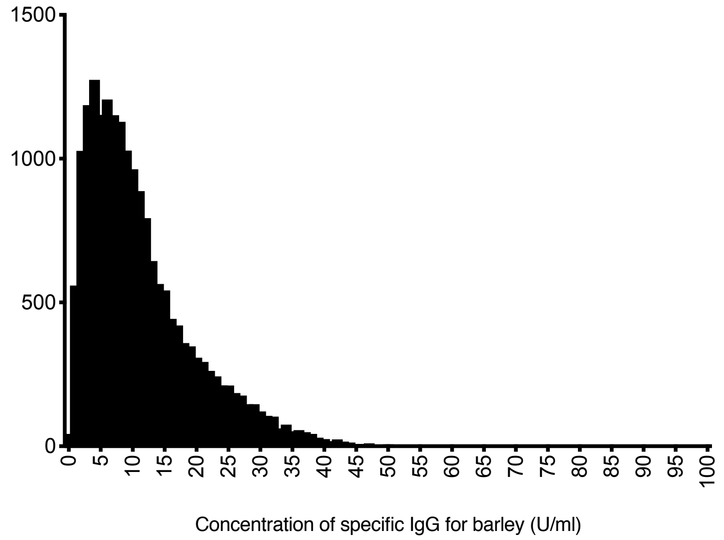

Frequency distribution of IgG for barley malt

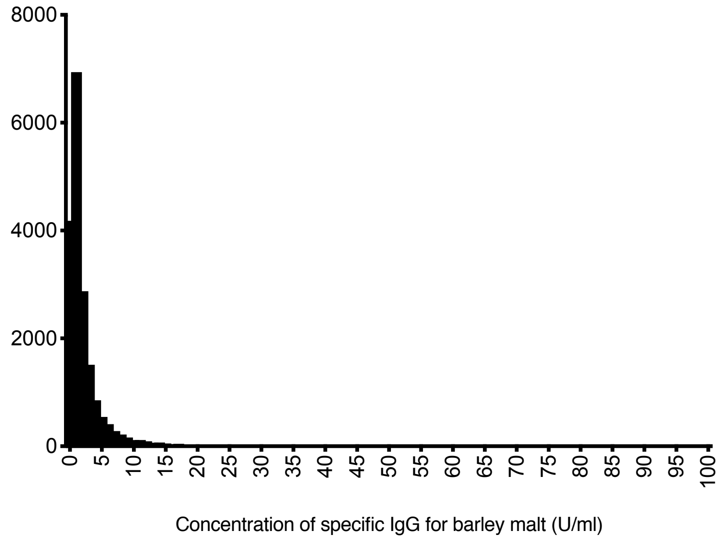

Frequency distribution of IgG for buckwheat

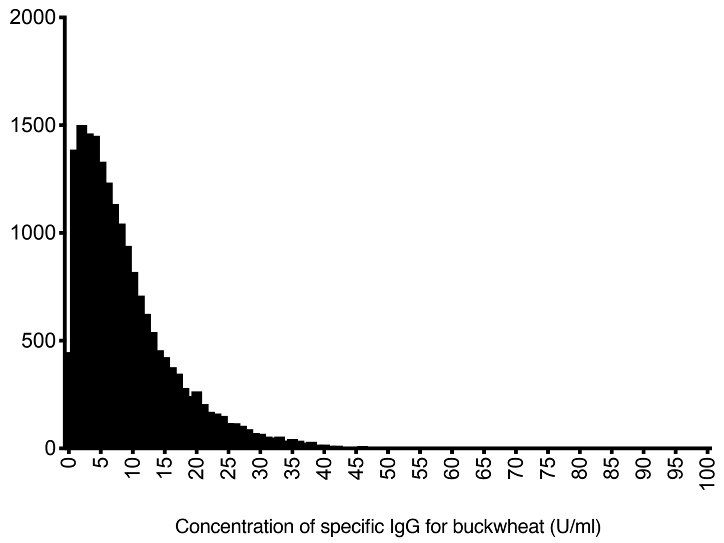

Frequency distribution of IgG for *Candida albicans*

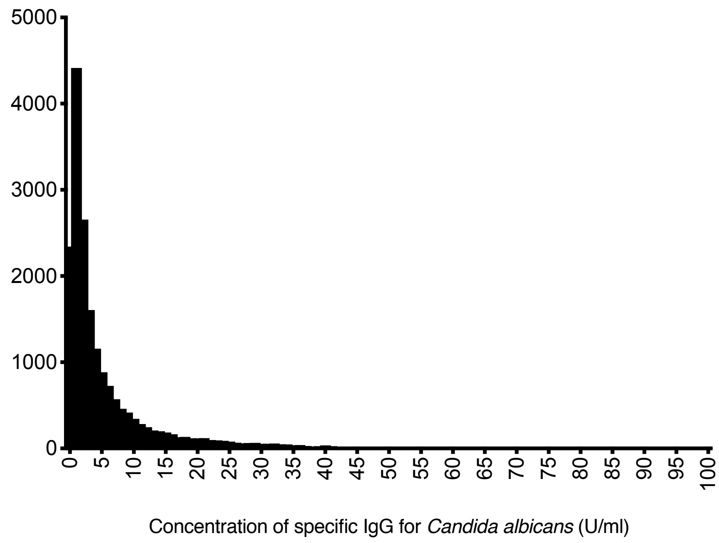

Frequency distribution of IgG for canned tuna

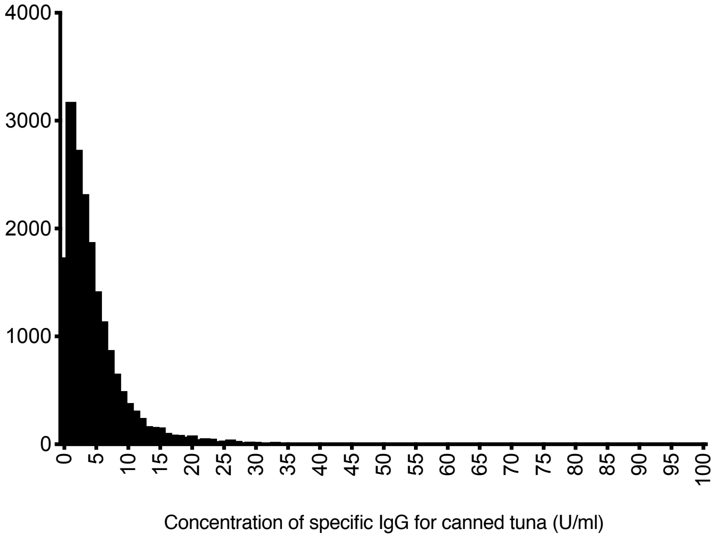

Frequency distribution of IgG for champignon mushrooms

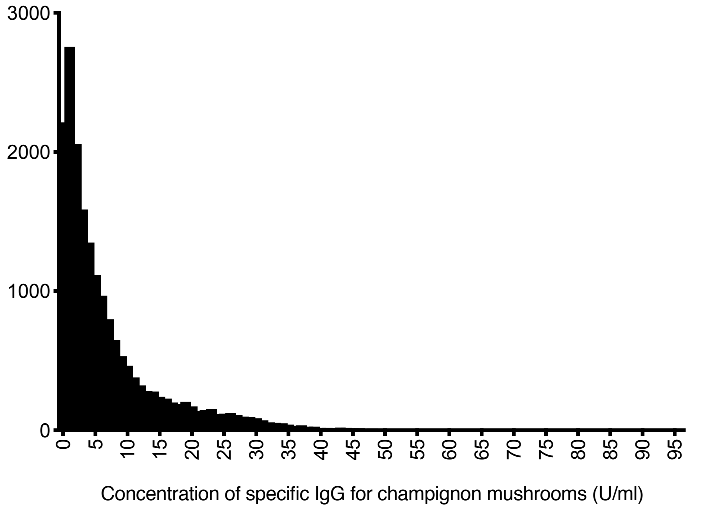

Frequency distribution of IgG for chicken

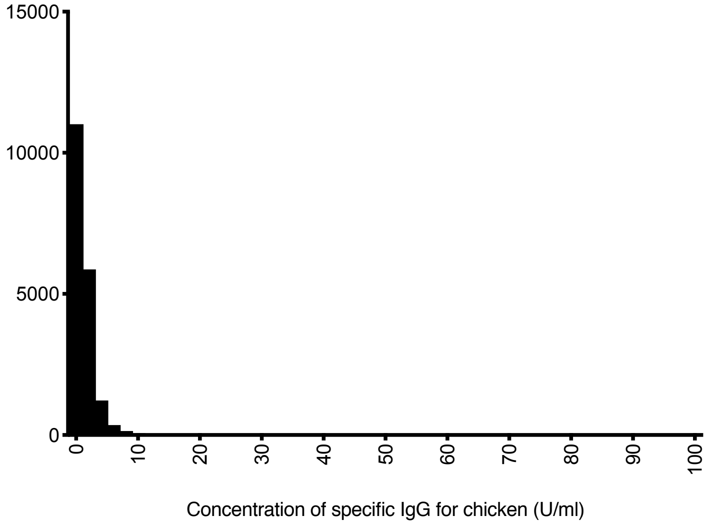

Frequency distribution of IgG for corn

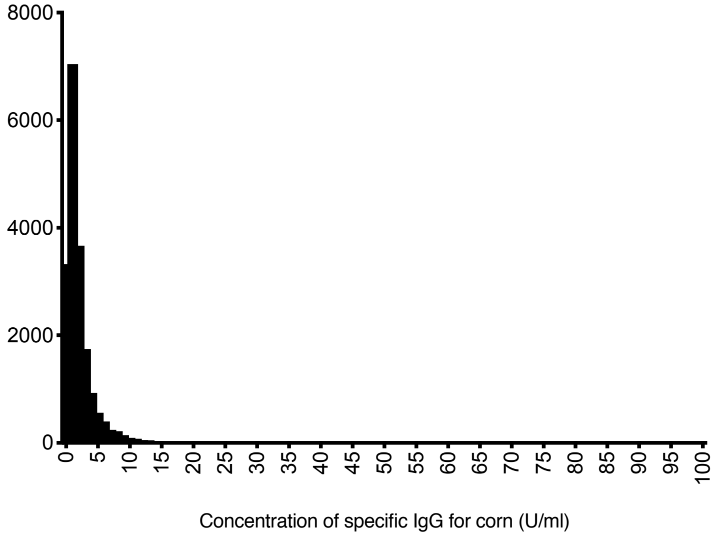

Frequency distribution of IgG for red grape

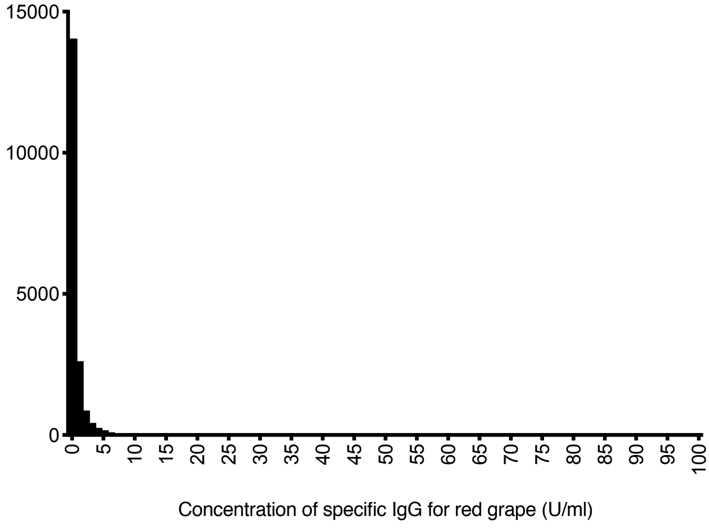

Frequency distribution of IgG for goat milk

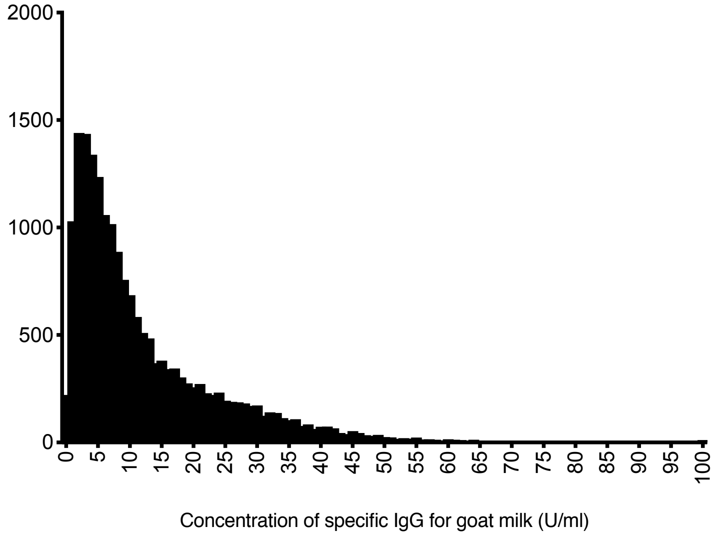

Frequency distribution of IgG for hazelnut

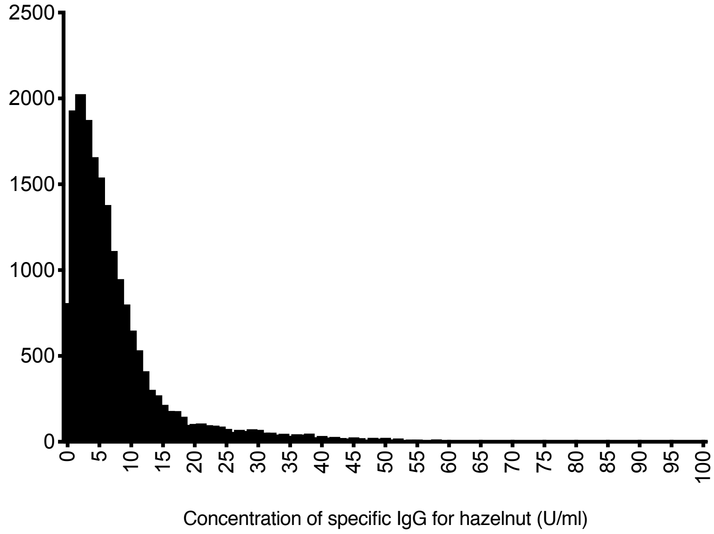

Frequency distribution of IgG for honey

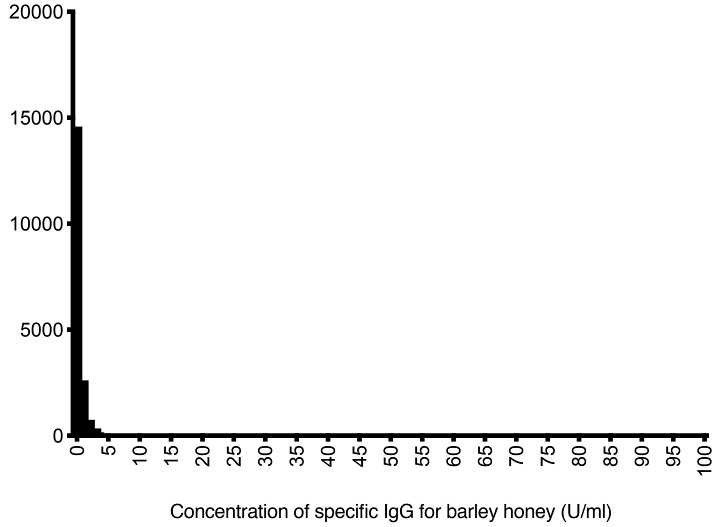

Frequency distribution of IgG for kamut

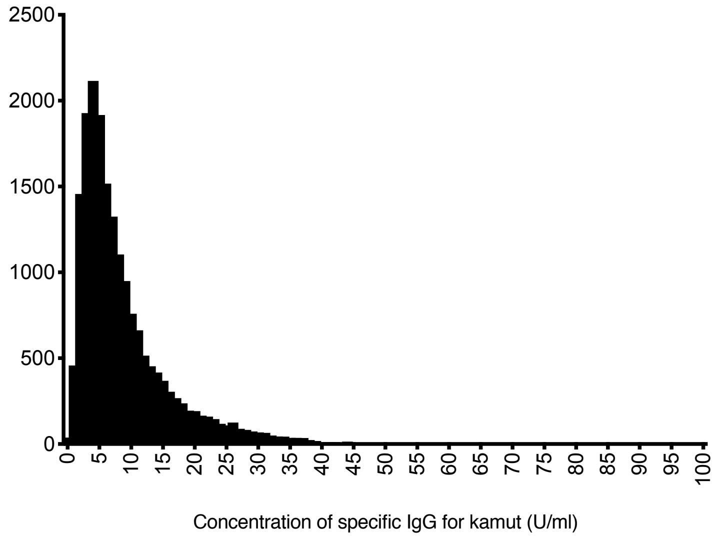

Frequency distribution of IgG for kiwi

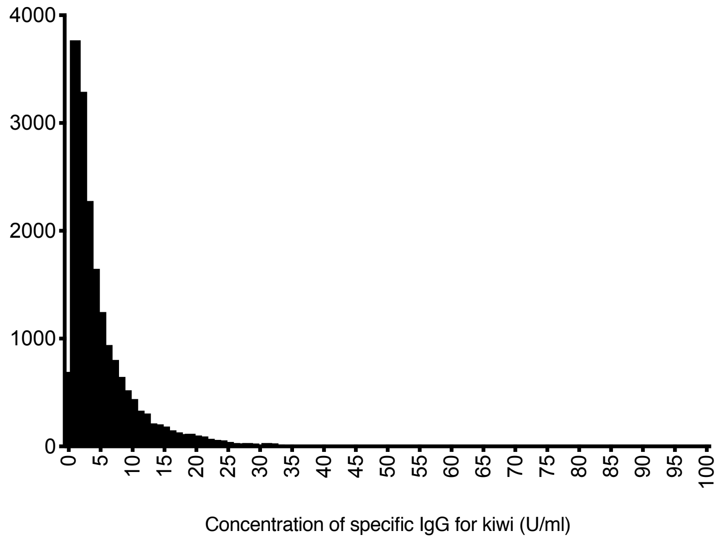

Frequency distribution of IgG for lentil

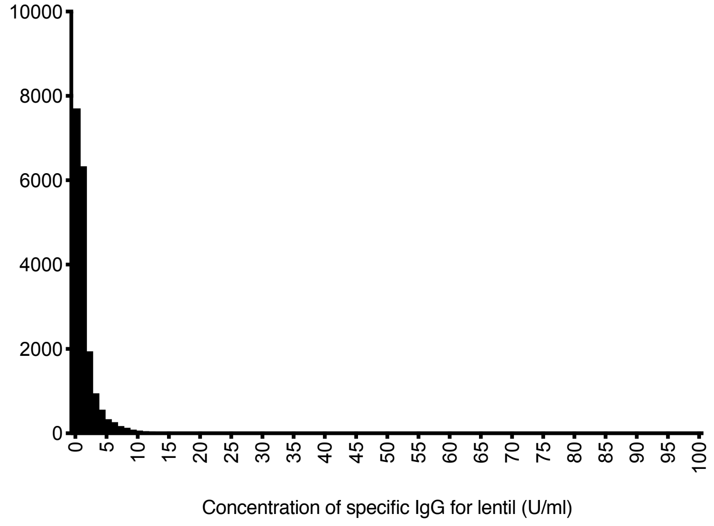

Frequency distribution of IgG for mozzarella cheese

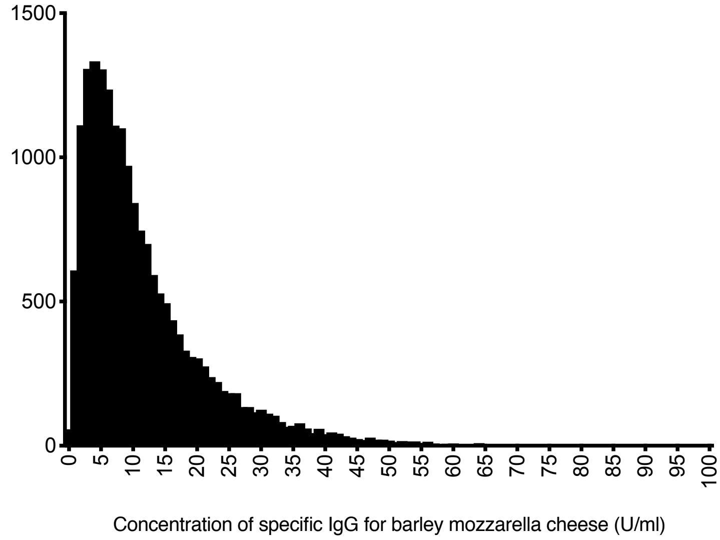

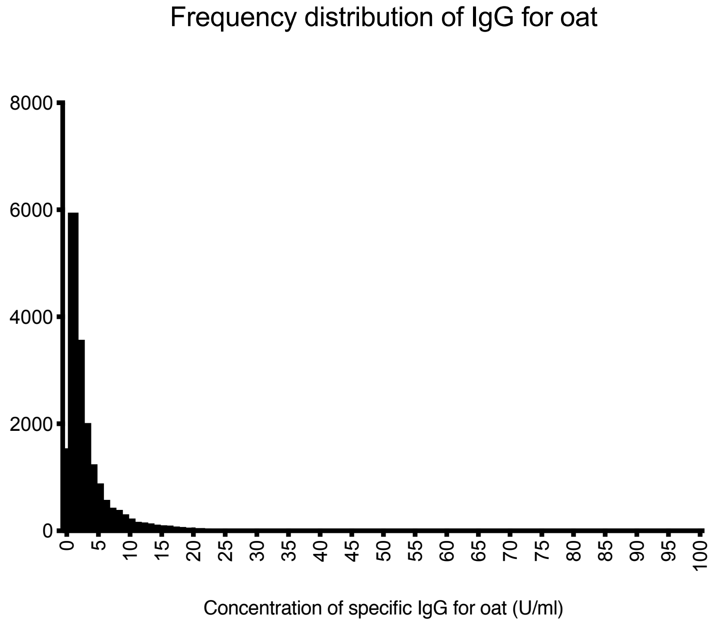
Frequency distribution of IgG for oat
0
2000
4000
6000
8000
0 5 10 15 20 25 30 35 40 45 50 55 60 65 70 75 80 85 90 95 100
Concentration of specific IgG for oat (U/ml)

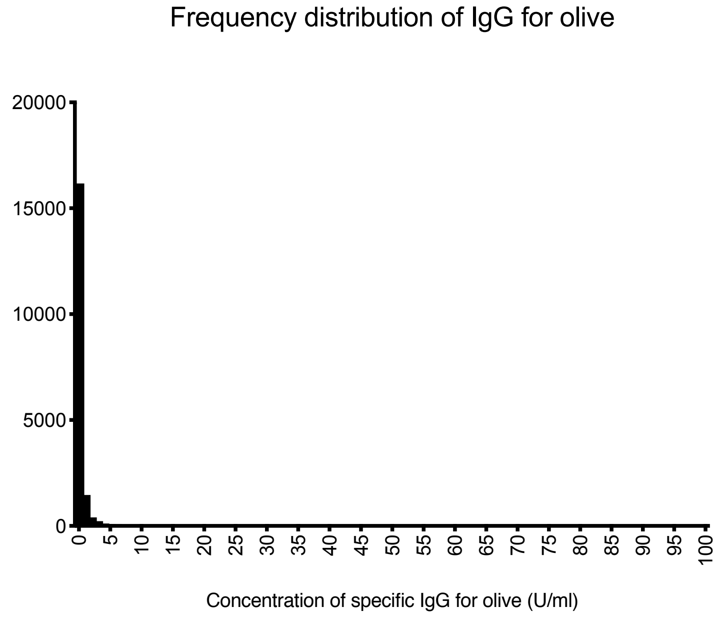
Frequency distribution of IgG for olive
0
5000
10000
15000
20000
0 5 10 15 20 25 30 35 40 45 50 55 60 65 70 75 80 85 90 95 100
Concentration of specific IgG for olive (U/ml)

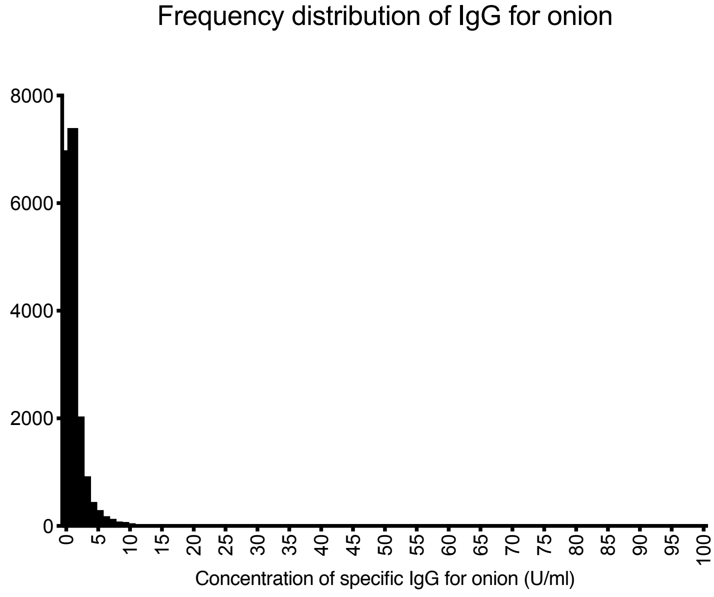
Frequency distribution of IgG for onion
0
2000
4000
6000
8000
0 5 10 15 20 25 30 35 40 45 50 55 60 65 70 75 80 85 90 95 100
Concentration of specific IgG for onion (U/ml)

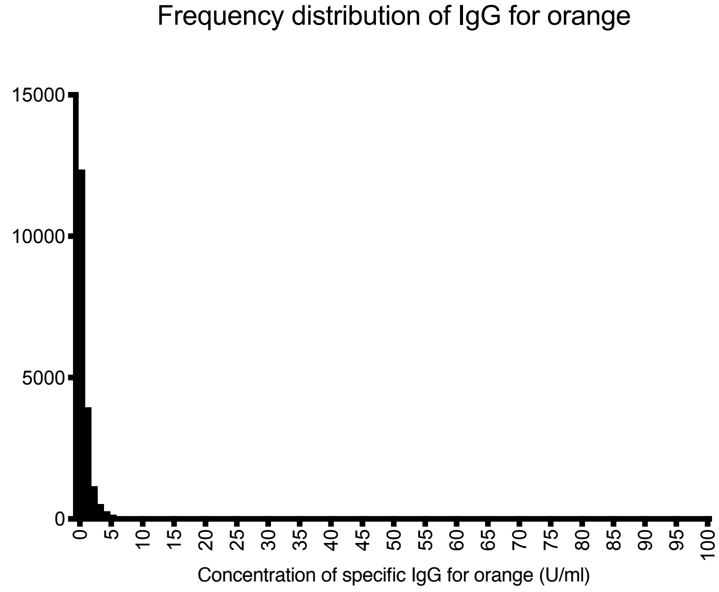
Frequency distribution of IgG for orange
0
5000
10000
15000
0 5 10 15 20 25 30 35 40 45 50 55 60 65 70 75 80 85 90 95 100
Concentration of specific IgG for orange (U/ml)

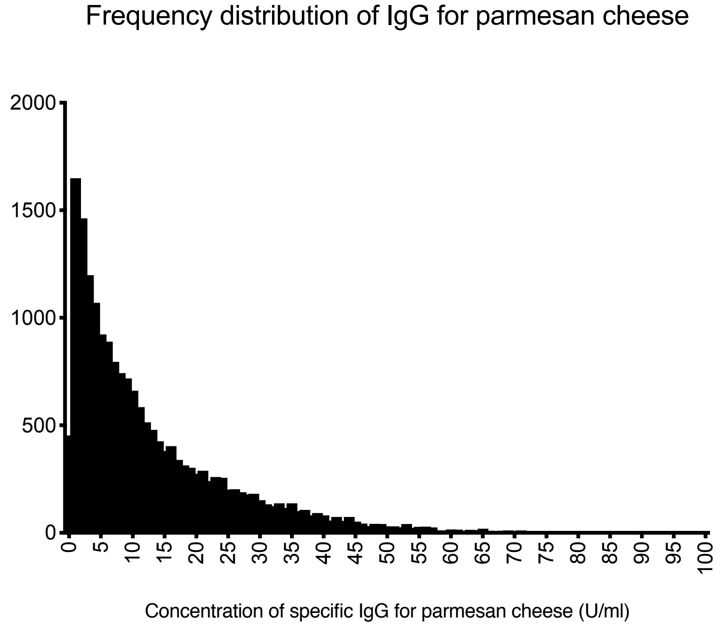
Frequency distribution of IgG for parmesan cheese
0
500
1000
1500
2000
0 5 10 15 20 25 30 35 40 45 50 55 60 65 70 75 80 85 90 95 100
Concentration of specific IgG for parmesan cheese (U/ml)

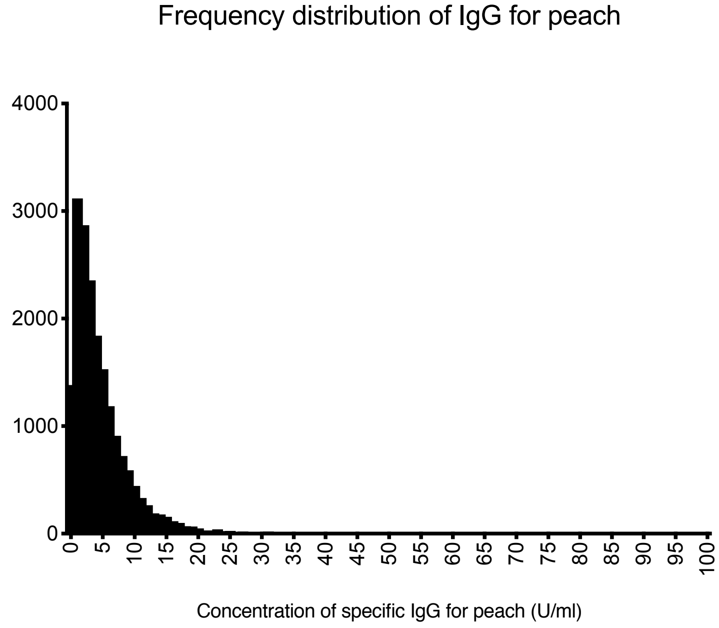
Frequency distribution of IgG for peach
0
1000
2000
3000
4000
0 5 10 15 20 25 30 35 40 45 50 55 60 65 70 75 80 85 90 95 100
Concentration of specific IgG for peach (U/ml)

Frequency distribution of IgG for porcini mushrooms

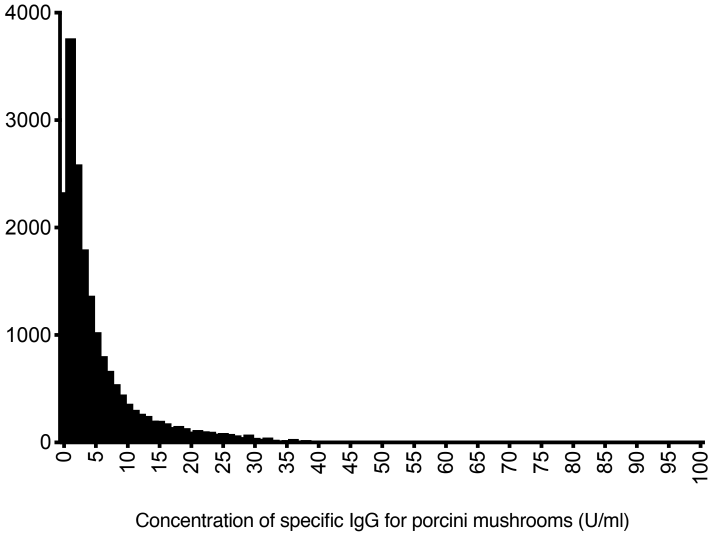

Frequency distribution of IgG for potato

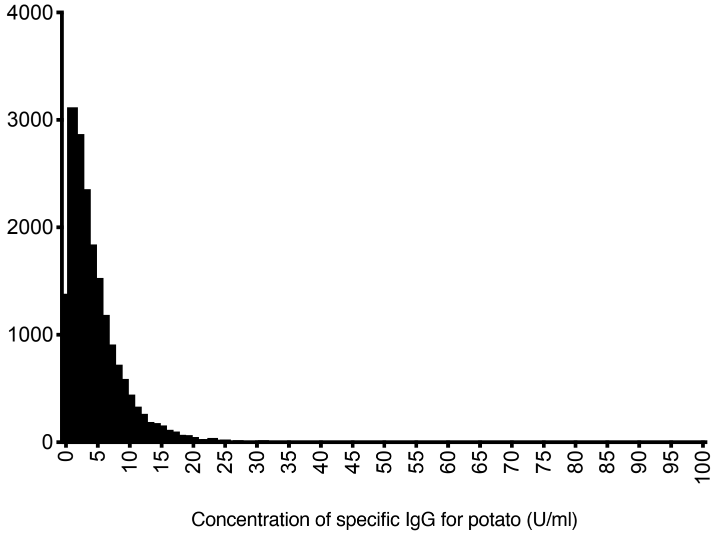

Frequency distribution of IgG for red grape

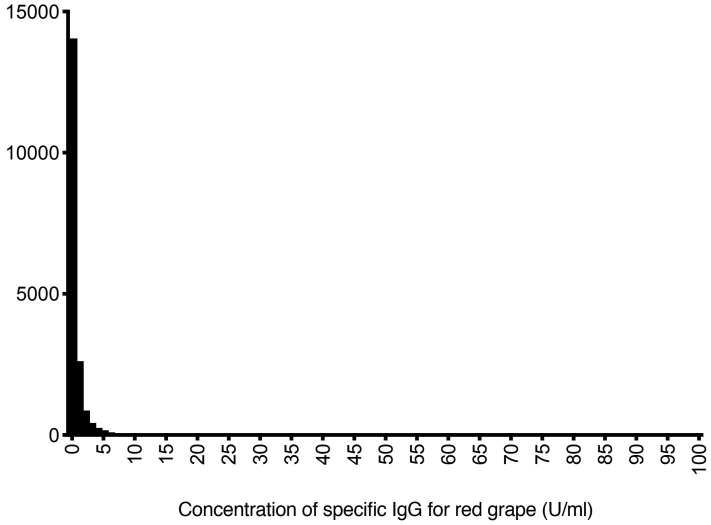

Frequency distribution of IgG for rice

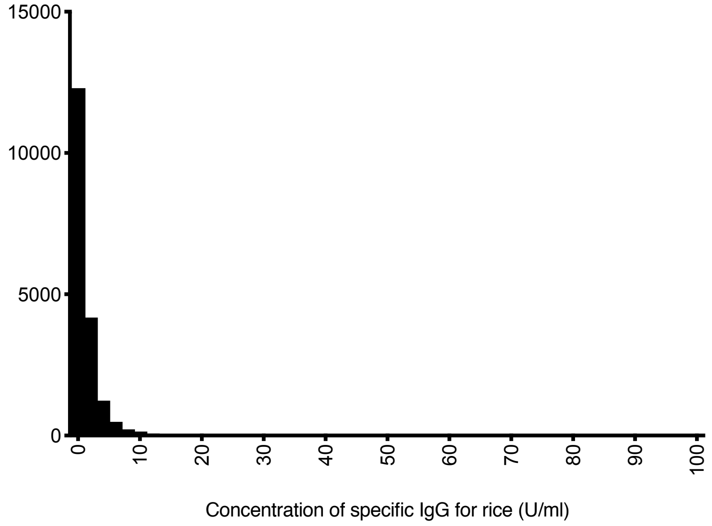

Frequency distribution of IgG for ricotta cheese

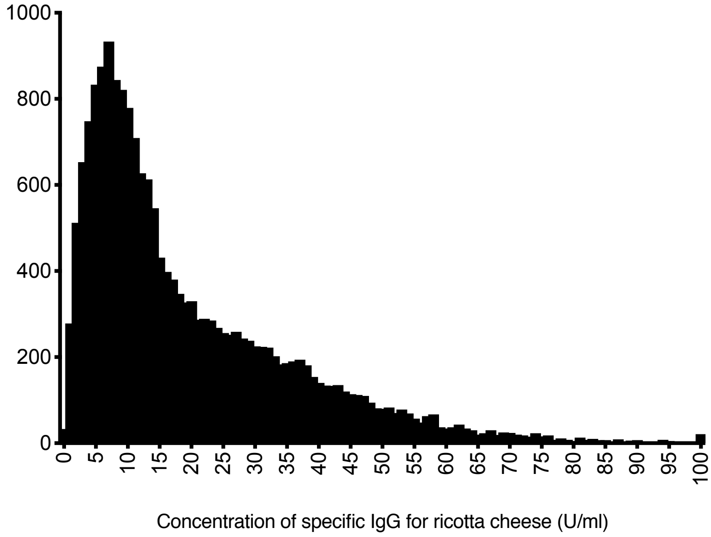

Frequency distribution of IgG for rye

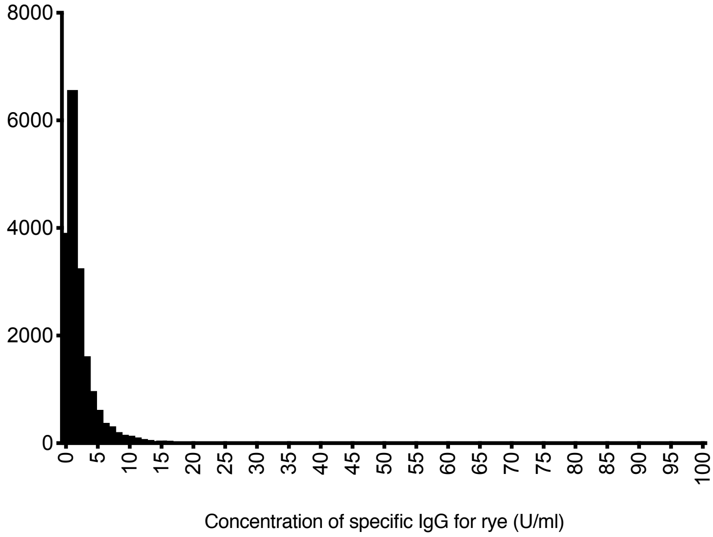

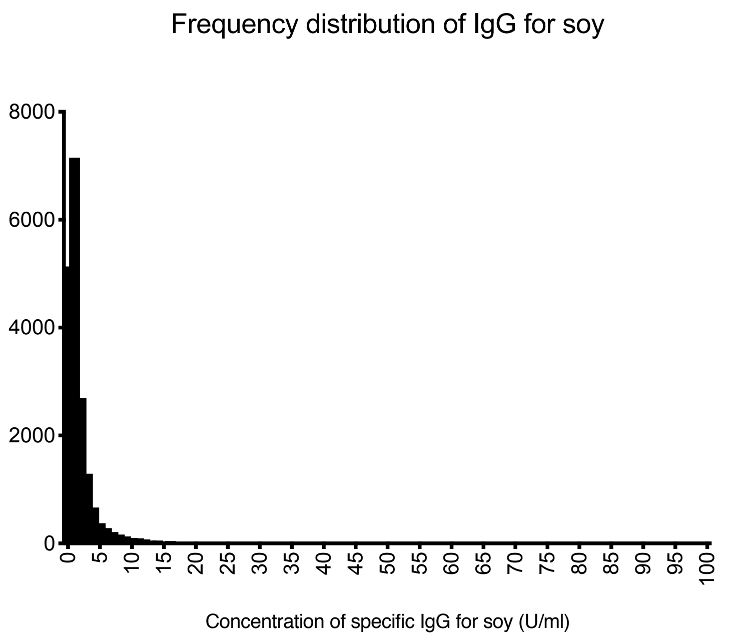
Frequency distribution of IgG for soy
8000
6000
4000
2000
0
0 5 10 15 20 25 30 35 40 45 50 55 60 65 70 75 80 85 90 95 100
Concentration of specific IgG for soy (U/ml)

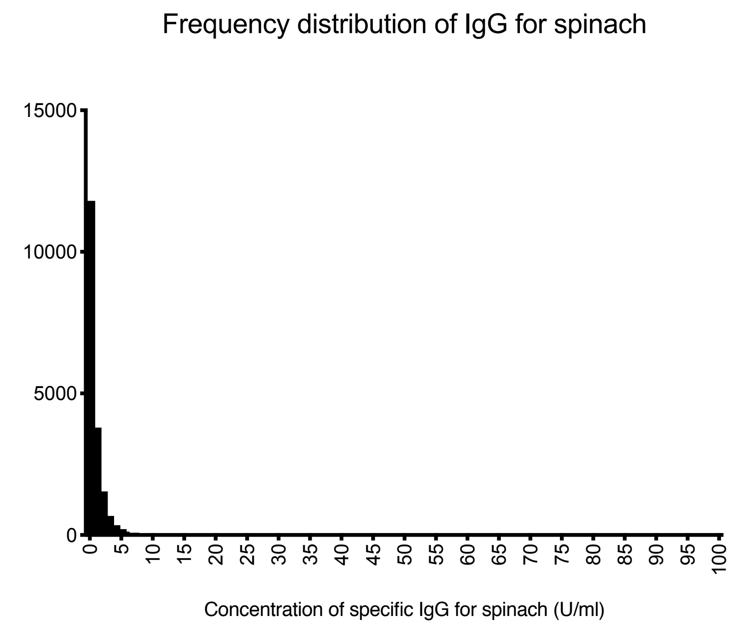
Frequency distribution of IgG for spinach
15000
10000
5000
0
0 5 10 15 20 25 30 35 40 45 50 55 60 65 70 75 80 85 90 95 100
Concentration of specific IgG for spinach (U/ml)

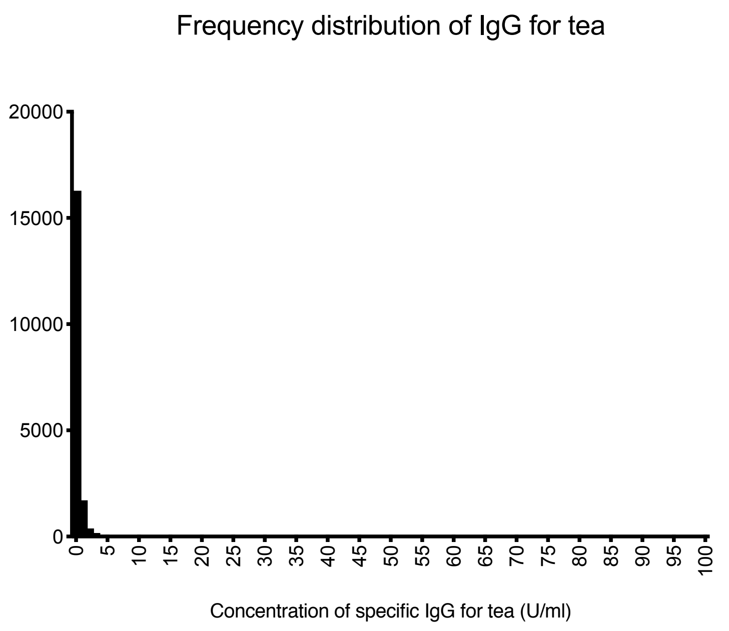
Frequency distribution of IgG for tea
20000
15000
10000
5000
0
0 5 10 15 20 25 30 35 40 45 50 55 60 65 70 75 80 85 90 95 100
Concentration of specific IgG for tea (U/ml)

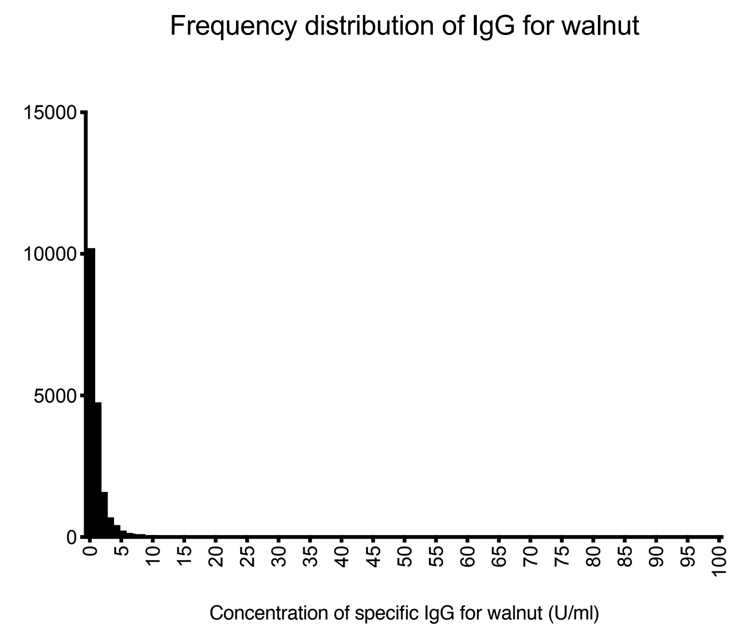
Frequency distribution of IgG for walnut
15000
10000
5000
0
0 5 10 15 20 25 30 35 40 45 50 55 60 65 70 75 80 85 90 95 100
Concentration of specific IgG for walnut (U/ml)

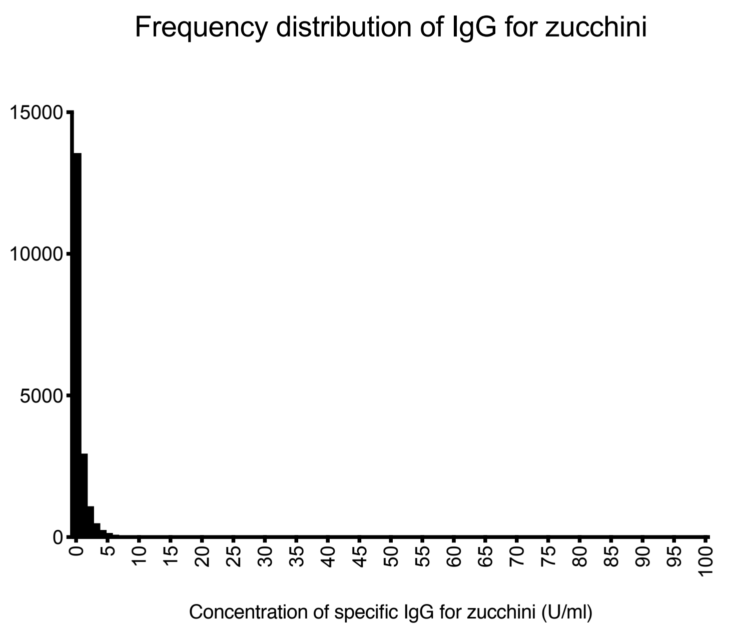
Frequency distribution of IgG for zucchini
15000
10000
5000
0
0 5 10 15 20 25 30 35 40 45 50 55 60 65 70 75 80 85 90 95 100
Concentration of specific IgG for zucchini (U/ml)

Frequency distribution of IgG for beer yeast

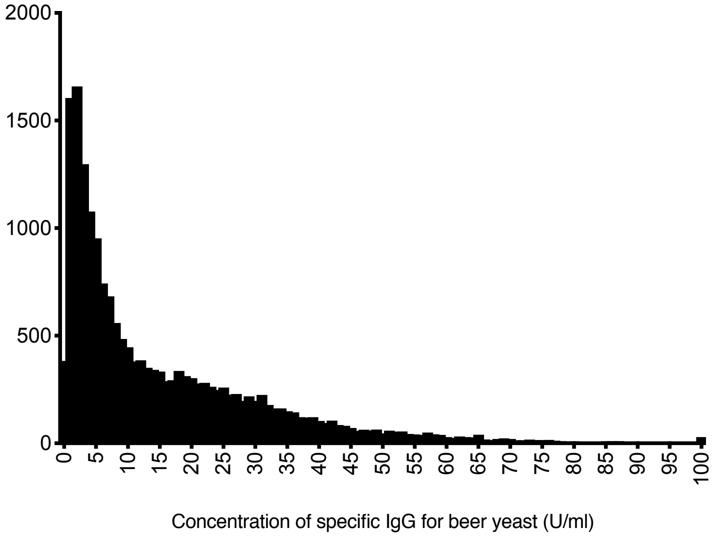

Frequency distribution of IgG for durum wheat

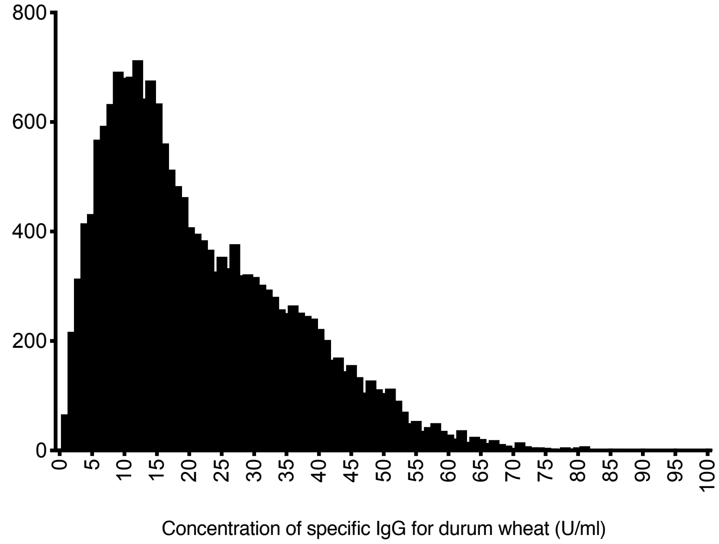

Frequency distribution of IgG for pork

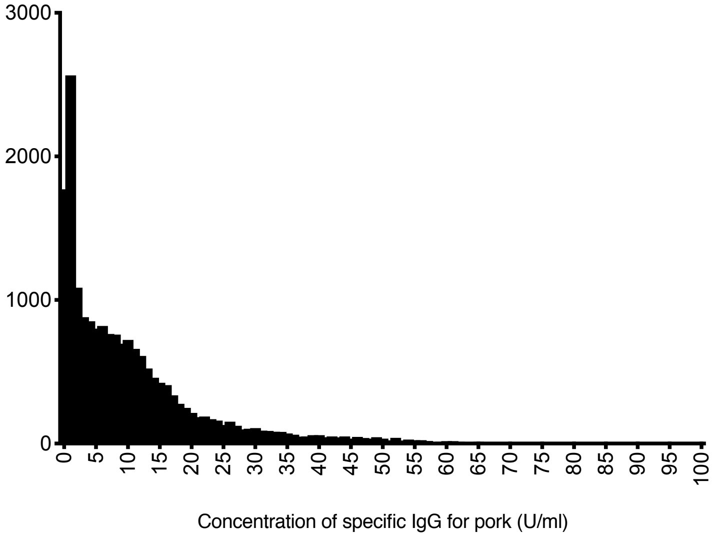

Frequency distribution of IgG for processed cheese

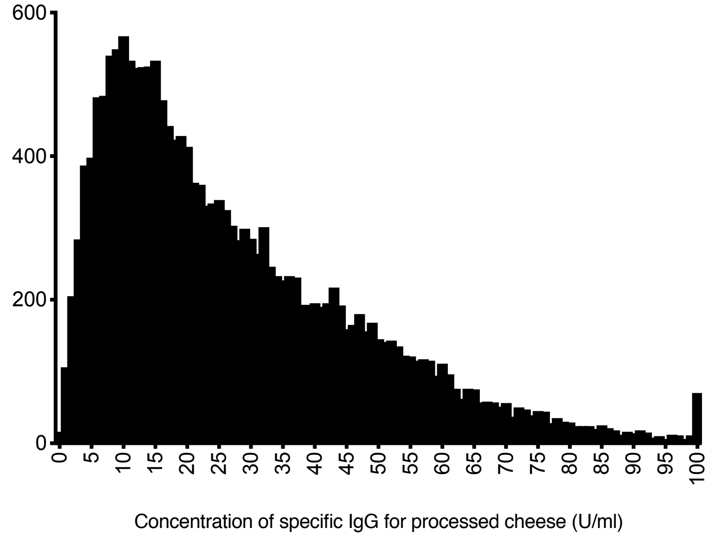

Frequency distribution of IgG for spelt

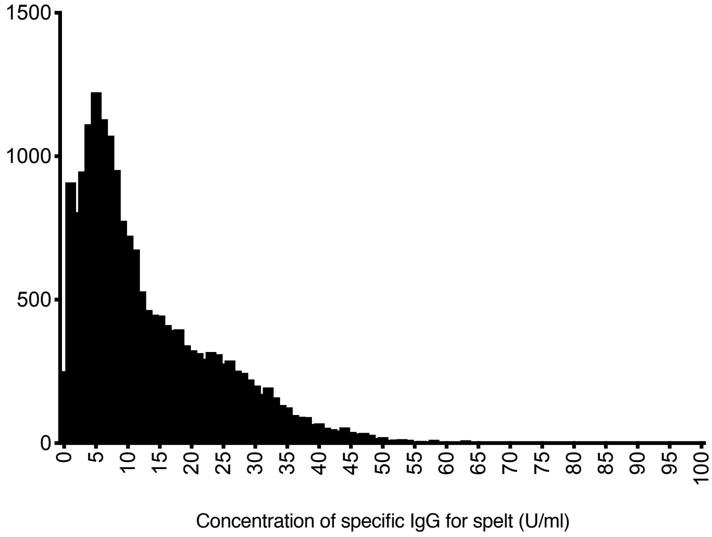

Supplement: Supplementary file 1 [file nutrients-11-01096-s001.pdf]
